# *Compañeros*: High School Students Mentor Middle School Students to Address Obesity Among Hispanic Adolescents

**DOI:** 10.5888/pcd14.170130

**Published:** 2017-10-12

**Authors:** Katherine R. Arlinghaus, Jennette P. Moreno, Layton Reesor, Daphne C. Hernandez, Craig A. Johnston

**Affiliations:** 1 Department of Health and Human Performance, University of Houston, Houston, Texas; 2 USDA/ARS Children’s Nutrition Research Center, Department of Pediatrics, Baylor College of Medicine, Houston, Texas

## Abstract

**Introduction:**

*Promotoras*, Hispanic community health workers, are frequently employed to promote health behavioral change with culturally bound Hispanic lifestyle behaviors. Peer health mentors have been used in schools to promote healthy nutrition and physical activity behaviors among students. This study investigates the efficacy of combining these 2 approaches by training high school health mentors, called *compañeros*, to engage Hispanic middle school students in a school-based obesity intervention as a strategy to promote and sustain reductions in standardized body mass index (zBMI).

**Methods:**

High school *compañeros* were trained to participate in a 6-month obesity program alongside middle school students in Houston, Texas. Middle school students were randomized to participate in the program either with *compañeros* (n = 94) or without *compañeros* (n = 95). The intervention was conducted from 2013 through 2016 in 3 cohorts of students, 1 each school year. Students were followed for 12 months. The primary outcome was zBMI, which was analyzed at baseline, 6 months, and 12 months.

**Results:**

Significant differences were found between conditions across time (*F* = 4.58, *P* = .01). After the 6-month intervention, students in the condition with *compañeros* had a larger decrease in zBMI (*F *= 6.94, *P* = .01) than students in the condition without *compañeros*. Furthermore, students who received the intervention with *compañeros* showed greater sustained results at 12 months (*F *= 7.65, *P* = .01).

**Conclusion:**

Using high school *compañeros* in an obesity intervention for Hispanic middle school students could be effective in promoting and maintaining reductions in zBMI.

**Editor’s Note:** This article is 1 of 2 winners of *PCD*‘s 2017 Student Research Paper Contest in the Doctoral category.

## Introduction

Although one of the strengths of school-based interventions for obesity is the ability to reach racial/ethnic minority groups who are at elevated risk, the success of school-based weight management interventions is not equivalent across races/ethnicities, and few obesity intervention programs exist that are tailored for racial/ethnic minority groups (1,2). A cost-effective public health strategy frequently used in Hispanic communities is to train community health workers, called *promotoras,* to promote healthy lifestyle behaviors (3,4). *Promotoras* are familiar with the population they serve and are typically well-respected members of the target community. These factors enable them to communicate health messages in a relatable way (5). Adapting the *promotoras* model to the middle school setting by training high school students as health mentors, called *compañeros*, may be one strategy to more effectively tailor weight management interventions for Hispanic adolescents.

Peer perception of lifestyle behaviors is important to adolescents (6), and evidence is beginning to establish teenagers as effective health mentors (7). However, few studies have assessed anthropometric measurements as an outcome (8–10). Of those that have, none were conducted with low-income, Hispanic adolescents, and none included a follow-up after the intervention to determine whether results were sustained. Our study aimed to examine whether the assistance of *compañeros* in the implementation of nutrition and physical activity lessons could be an effective strategy for delivering an obesity prevention program to middle school students in a predominantly Hispanic school system.

## Methods

Sixth-grade and seventh-grade students (n = 506) were recruited from a charter school in Houston, Texas, that serves students in grades 6 through 12. Although all students who provided verbal assent and had parental consent were given the opportunity to participate in the intervention, only those who were overweight or obese (n = 189), defined as having a body mass index (BMI, kg/m^2^) at or above the 85th percentile for age and sex according to the guidelines of the Centers for Disease Control and Prevention (CDC) (11) were included in this analysis. This sample size satisfied the 200 participants (100 in each condition) that were calculated to be needed to have an 80% likelihood of detecting a 0.09-unit difference in zBMI (standardized BMI) between conditions. The power analysis assumed nominal values for type I and type II error rates (5% and 20% respectively; 2-tailed) and an attrition rate of 20%. Students were randomized to receive either an obesity intervention with *compañeros* (n = 94) or without *compañeros* (n = 95). All students self-identified as Hispanic.

### Study design

Participants in both conditions received an obesity intervention for 50 minutes, 5 days a week, for 6 months during students’ physical education (PE) class period. The intervention was conducted from 2013 through 2016 in 3 cohorts, 1 each school year, and participants were followed for 12 months. Because of the school calendar, the intervention was interrupted by various school breaks. To prevent contamination, students’ schedules were developed before the beginning of the school year so that all students randomized to a particular condition were in classes only with students who were also randomized to the same condition. Interventions were led by PE teachers who were trained by research staff members as described elsewhere (12). Each week, the students participated in 1 day of healthy eating activities and 4 days of physical activity. This program was based on a school-based obesity intervention with demonstrated efficacy among this population (13,14). Details about the intervention and curriculum are available elsewhere (12,14,15). In addition to the physical activity and nutrition components, the intervention included behavioral modification through a token economy system in which the students received points for participation that they could accumulate and redeem for prizes. The only difference between the 2 conditions was the presence or absence of *compañeros*.

High school students were selected to be *compañeros* if they met the following criteria: were recommended by a teacher, had an opening in their schedule during intervention periods, and expressed a desire to be involved. Weight was not a criterion for either *compañeros* or middle school students to participate in the study. *Compañeros* and middle school students were not matched by weight or racial/ethnic characteristics. However, because the school has a predominantly Hispanic student body, all *compañeros* and middle school students were Hispanic. In this school district, high school and middle school students were taught in the same building.

*Compañeros* meeting criteria were trained daily for 2 weeks on how to lead all of the intervention activities. This training approach was similar to that used to train the PE teachers (12). The training curriculum mirrored the intervention curriculum, included basic nutrition and physical activity education, and was designed to help *compañeros* identify strengths and weaknesses in their own diets and physical activity habits. Training provided *compañeros* with ideas to use when talking with middle school students about how to make improvements in their diets and activity behaviors. *Compañeros* were trained on each intervention activity until they were able to perform each themselves and explain to others how to do it. *Compañeros* were provided with conversation starters and practiced initiating conversations about the curriculum with peers. Lastly, *compañeros* were trained in how to provide praise and the importance of modeling. *Compañeros* were considered to be proficient in this activity when they were able to demonstrate the use of praise correctly in 3 different student scenarios.

Once trained, *compañeros* were instructed to engage in intervention activities with the middle school students. Before each class, the PE teacher informed *compañeros* of the topic of focus for the day (eg, strategies to eat more vegetables, ways to be more active throughout the day). During class, *compañeros* were to initiate a discussion of the selected topic with their group of middle school studentss. For example, between exercise stations *compañeros* might talk about what they were going to eat for lunch that day or discuss their favorite vegetables. PE teachers regularly met with *compañeros* to provide feedback on how they were doing and give guidance as needed.

In the without *compañeros* condition, all variables were held constant between conditions with the exception of the *compañeros* component. A trained PE teacher provided the same lessons as with the *compañeros* condition. The only difference was that they conducted class without *compañeros* assistance.

Researchers monitored the implementation fidelity of each condition. For both conditions, researchers recorded the number of nutrition and physical activity sessions conducted. They also randomly assessed 10% of classes to record how frequently the PE teacher provided positive reinforcement and constructive feedback to students. Weekly meetings were conducted with the PE teacher to discuss issues related to intervention adherence. The fidelity check process was the same for both conditions except that in the *compañeros* condition, the implementation of fidelity of the *compañero* role was also monitored. Specifically, researchers randomly observed 10% of classes to record how frequently *compañeros* modeled healthy behavior and provided praise to the middle school students.

### Measures

Middle school students’ height and weight were regularly measured throughout the study. Baseline, 6-month, and 12-month assessments were included in this analysis. At each assessment point, height was measured without footwear using a SECA 213 stadiometer (SECA). Weight was assessed in light clothing and without footwear using a Tanita BWB-800 digital scale (Tanita Corp). BMI was calculated from students’ weight and height. BMI percentiles were determined by using the students’ age and sex and were classified according to CDC guidelines (11). BMI percentiles were standardized to sex and age norms to determine zBMI.

The interpretation of height and weight for adolescents is complicated because adolescents are growing and developing. To enable a more comprehensive interpretation of anthropometric changes in adolescents, zBMI, BMI percentile, and BMI were included as outcomes. The primary outcome was zBMI, because the use of this metric is standard practice in research (16). Both zBMI and BMI percentiles account for age, sex, and the expected growth and development of adolescents. Possibly because pediatricians often speak to parents about their child’s growth in terms of percentiles, the meaning of BMI percentile is more interpretable for a larger audience than the meaning of zBMI. Although zBMI is more sensitive than BMI percentile, neither of these metrics is sensitive to change at extreme ranges, such as that indicative of extreme obesity. BMI was included as an outcome to overcome this shortcoming because, although BMI does not account for age, sex, or the expected growth of adolescents, its sensitivity does not diminish at ranges suggestive of extreme obesity.

### Data analysis

Statistical analyses were performed using SPSS, version 19.0 (SPSS, Inc); χ^2^ and independent samples *t* tests were conducted to compare differences between conditions at baseline and between those with and without measures at 6 and 12 months. A 2 × 3 repeated measures analysis of covariance (ANCOVA) was used to determine differences in weight outcomes between conditions across all periods. Post-hoc analyses (2 × 2 repeated measures ANCOVA) were conducted at both 6 and 12 months. To be consistent with the Consolidated Standards of Reporting Trials 2010 Statement (17), in addition to the model developed for the main analysis, the last observation carried forward (LOCF) method was used to create an intention-to-treat model to include those without 6-month or 12-month measurements. This method replaces missing data with the data most recently collected. Mean change scores for height, weight, BMI, BMI percentile, and zBMI were computed for each condition, from baseline to 6 months and from baseline to 12 months for both the main analysis and the intention-to-treat analysis. This study was approved by the Institutional Review Board for Human Subjects at the Baylor College of Medicine.

## Results

**Figure 1:**
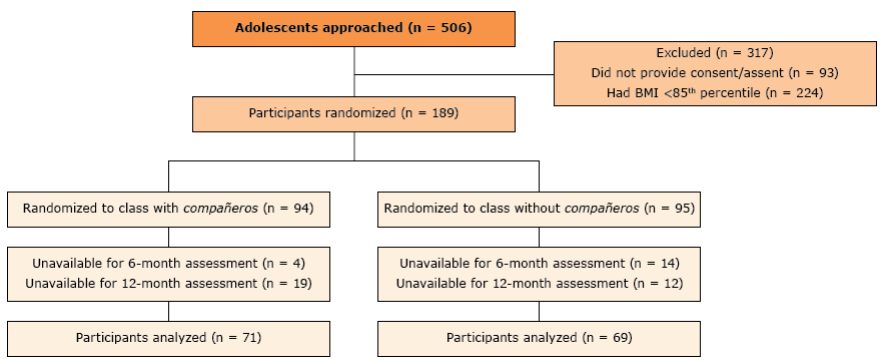
CONSORT diagram illustrating the flow of participants through the study, an obesity prevention intervention using *compañeros*, Houston, Texas, 2013–2016. Participants included in the main analysis had baseline, 6-month, and 12-month assessment data. Abbreviation: CONSORT, the Consolidated Standards of Reporting Trials.

Of the 189 students initially included in the study (94 in the *compañeros* condition and 95 in the without *compañeros* condition), 140 were available for assessment at 6 and 12 months, 71 students in the with *compañeros* condition and 69 in the without *compañeros* condition (Figure 1). The 49 students who were unavailable for assessment were excluded from our main assessment. No significant differences in age, sex, height, weight, or BMI were observed at baseline between conditions (Table 1). There was a 74.1% retention rate at 12 months (n = 140 students remained). Attrition did not differ significantly among the 71 students remaining in the *compañeros* condition (24.5% attrition) and the 69 students remaining in the condition without *compañeros* (27.4% attrition). Students excluded from analysis (those unavailable for measurements at both 6 and 12 months) had a higher initial weight, BMI, and zBMI than did those whom we were able to assess at each time point (Table 1). Because of this, baseline weight was used as a covariate during all analyses.

**Table 1:** Comparison of Baseline Characteristics of Participants (N = 189), by 12-month Attrition and by Treatment Condition^a^, *Compañeros* Obesity Intervention, Houston, Texas 2013–2016

Characteristic	Included in Main Analysis	Excluded From Main Analysis^b^	*P* Value^c^	With *Compañeros* Condition	Without *Compañeros* Condition	*P* Value^d^
Total, n	140	49	—	94	95	—
Age, y	13.02 (0.56)	12.90 (0.56)	.17	12.91 (0.48)	12.94 (0.63)	.71
Female, n (%)	66 (47)	31 (63)	.07	48 (51)	49 (52)	—
Height, cm	157.93 (6.67)	158.10 (7.25)	.88	157.54 (6.97)	158.57 (7.20)	.32
Weight, kg	65.68 (9.30)	69.92 (13.83)	.02	68.32 (13.04)	69.31 (12.84)	.60
BMI, kg/m^2^	26.30 (3.10)	27.86 (4.56)	.01	27.40 (4.03)	27.51 (4.53)	.85
zBMI	1.64 (0.37)	1.81 (0.45)	.01	1.78 (0.41)	1.76 (0.46)	.77
BMI percentile	93.86 (3.97)	95.13 (4.04)	.06	95.04 (3.82)	94.57 (4.27)	.43
Attrition at 12 mos, n (%)	0 (0)	49 (100)	—	23 (24.5)	26 (27.4)	.74

Abbreviations: —, not applicable; BMI, body mass index; zBMI, standardized BMI.

a Values are mean (standard deviation) unless otherwise noted.

b Participants randomized into a study condition were not included in the analysis if they were unavailable for both 6-month or 12-month assessments.

c *P* values were determined by an independent samples *t* test and χ^2^ tests between participants who were and were not included in the main analysis.

d *P* values were determined by independent samples *t* tests and χ^2 ^tests between with *compañeros* and without *compañeros* conditions.

Implementation fidelity was high overall for both conditions. In both conditions, all 24 nutrition sessions and 96 physical activity sessions were conducted, and PE teachers provided constructive feedback in 100% of the observed classes. PE teachers provided positive reinforcement in 90% of the observed classes in the *compañeros* condition and in 95% of the observed classes in the condition without *compañeros* condition. In the *compañeros* condition, *compañeros* modeled healthy behaviors in 98% of the observed classes and provided praise in 94% of the observed classes.

**Figure 2:**
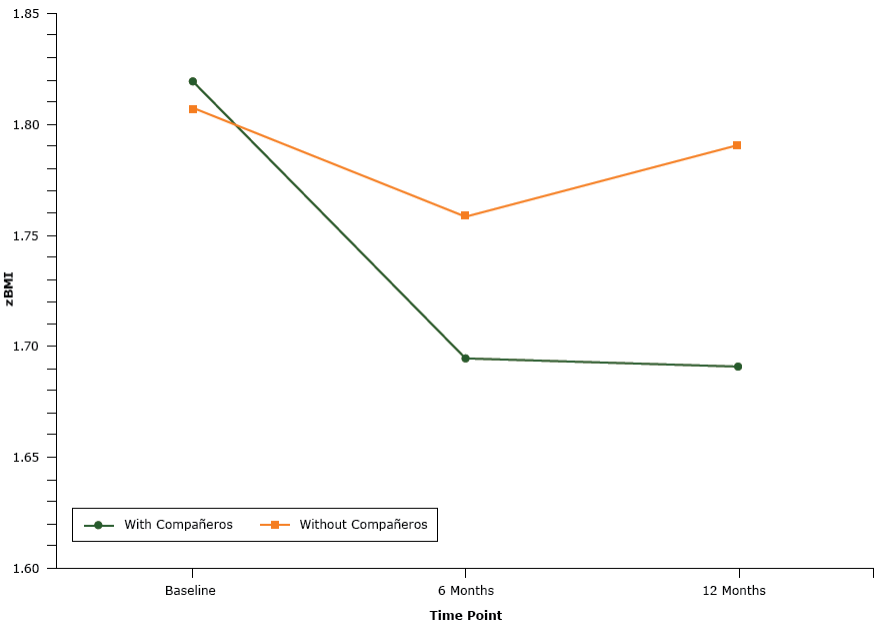
Comparison by study group of mean zBMI of participants at baseline, 6 months, and 12 months for participants in the with *compañeros* condition and participants in the without *compañeros* condition, an obesity prevention intervention using *compañeros*, Houston, Texas, 2013–2016. **Table:** 

Results from the ANCOVA analysis indicated that, compared with students in the condition without *compañeros*, students in the *compañeros* condition decreased their zBMI (*F *= 4.58, *P* = .01) (Figure 2).

Time	With *Compañeros*	Without *Compañeros*
	zBMI	
Baseline	1.82	1.81
6 months	1.70	1.76
12 months	1.69	1.79

Post hoc analyses from baseline to 6 months and baseline to 12 months indicated differences in zBMI between conditions (*F *= 6.94, *P* = .01 and *F *= 7.65, *P* = .01, respectively). Eighty percent of students in the *compañeros* condition and 64% of students in the condition without *compañeros* decreased or maintained zBMI from baseline to 6 months. At 12 months, 68% of students in the *compañeros* condition and 55% of students in the condition without *compañeros* had decreased or maintained zBMI from baseline.

BMI scores did not decrease for all outcome variables between conditions from baseline to 6 months and 12 months for either condition (Table 2). The mean change in BMI from baseline to 12 months was significantly different between conditions; zBMI and BMI percentile decreased from baseline to 6 months and from baseline to 12 months for both conditions. The *compañeros* condition had a significantly greater decrease in zBMI at both 6 months and 12 months than the condition without *compañeros*.

**Table 2:** Changes in Body Characteristics of Participants (N = 189) at 6 Months and 12 Months by, Treatment Condition^a^, *Compañeros* Obesity Intervention, Houston, Texas 2013–2016

Characteristic	With *Compañeros* (n = 71)	Without *Compañeros* (n = 69)	*P* Value^b^	With *Compañeros *(n = 94)	Without *Compañeros* (n = 95)	*P* Value^b^
	Main Analysis^a^, Mean (SD)			Intention-to-Treat^c^, Mean (SD)		
**Change in values from baseline to 6 months**						
Height, cm	2.25 (2.02)	2.62 (1.92)	.27	2.17 (1.95)	2.27 (1.96)	.73
Weight, kg	0.88 (2.92)	2.61 (3.88)	.001	1.18 (2.89)	2.03 (3.82)	.09
BMI, kg/m^2^	−0.42 (1.23)	0.13 (1.45)	.02	−0.27 (1.20)	0.03 (1.41)	.12
*z*BMI	−0.12 (0.18)	−0.05 (0.16)	.01	−0.10 (0.17)	−0.05 (0.16)	.04
BMI percentile	−1.67 (3.25)	−0.91 (2.94)	.15	−1.31 (2.93)	−0.83 (2.79)	.26
**Change in values from baseline to 12 months**						
Height, cm	4.37 (3.10)	3.82 (4.47)	.40	3.89 (2.94)	3.27 (4.06)	.24
Weight, kg	4.17 (5.55)	6.11 (4.63)	.03	4.05 (5.22)	4.77 (5.13)	.34
BMI	0.12 (1.99)	1.11 (2.19)	.01	0.25 (1.89)	0.78 (2.13)	.07
*z*BMI	−0.13 (0.26)	−0.01 (0.21)	.01	−0.10 (0.24)	−0.03 (0.21)	.02
BMI percentile	−1.86 (4.15)	−0.60 (3.09)	.05	−1.40 (3.80)	−0.88 (3.36)	.33

Abbreviations: BMI, body mass index; zBMI, standardized BMI.

a Participants with both 6-month and 12-month assessments.

b *P* values were determined by an independent samples *t* test between conditions.

c Students initially assigned to the 2 conditions who were unavailable for measurement assessments at 6 and 12 months. Analysis was conducted by using the last observation carried forward method. All participants who had been randomized to a study condition were included in this analysis.

As with the main analysis, the intention-to-treat ANCOVA showed that compared with students in the condition without *compañeros*, students in the *compañeros* condition had a significantly decreased zBMI (*F *= 3.27, *P* = .04). The change in zBMI between conditions for both 6- and 12-month post hoc analyses also showed significant differences between conditions (*F *= 5.08, *P* = .04; *F *= 5.62, *P* = .02, respectively).

## Discussion

The purpose of this randomized controlled trial was to see if the addition of *compañeros* to an established teacher-led, school-based obesity intervention (12) for middle school Hispanic students would be a more effective strategy for delivering the intervention than teachers delivering the intervention without *compañeros.* At both 6 months and 12 months, students in the *compañeros* condition had a significantly lower zBMI than those in the condition without *compañeros*. The paucity of school-based interventions for Hispanic adolescents makes it difficult to directly compare the findings of this study to other studies (18). However, the results of this study are consistent with obesity interventions for adolescents in general, in which zBMI has been estimated to decrease by less than 0.1 units from baseline to intervention end (19).

Mean weight, height, and BMI increased from baseline to 12 months in both conditions. This change is expected because adolescents are still growing. The goal of adolescent obesity interventions is not necessarily weight loss, but a slowed weight gain relative to height. The statistically smaller increase in BMI observed in the condition with *compañeros* compared with the condition without *compañeros* indicates that the presence of *compañeros* was more effective at changing the trajectory of weight gain relative to height.

Although school-based interventions have generally been able to create short-term reductions in zBMI, few have been able to accomplish maintenance of zBMI (19). Maintenance of results is particularly discouraging when intervention implementation is translated from research professionals to teachers and staff at a school (12). The results of this study are compelling because students who received the intervention with *compañeros* demonstrated greater maintenance in zBMI reduction at a year than those who received the intervention without *compañeros*. The addition of *compañeros* appears to be a possible solution to bridge the maintenance gap in the translation of intervention implementation from research professionals to a school’s teachers and staff.

One potential explanation for why the *compañeros* condition was more successful than the condition without *compañeros* is the possibility that *compañeros* were able to individually tailor the program for the middle school students in a way PE teachers were unable to. This suggestion is consistent with hypothesized reasons for the success of *promotoras* in community-based programs. As members of the community that they serve, *promotoras* are able to relate to program participants in a way medical professionals are often unable to (5). Because *compañeros* attended the same school and had similar socioeconomic and racial/ethnic backgrounds as the middle students, they likely had a fuller understanding of the middle students’ school, familial, and social environments. Although no data were collected to determine how middle school students perceived *compañeros*, the endorsement of healthy behaviors by high students, who are thought to be respected and admired by middle students, likely contributed to intervention engagement and sustained behavior change (20). Another plausible explanation for the differences seen between the 2 conditions is that students who received the intervention with *compañeros* received more attention, and this additional attention could have contributed to improved outcomes.

Because of the population of our study (ie, low income, Hispanic adolescents attending a charter school), additional research is needed to determine the generalizability of this type of intervention in other settings. However, the strategy of using peers to promote and sustain weight outcomes is likely generalizable to a variety of populations. For example, findings from this study are consistent with those of peer health mentoring interventions with Appalachian youths (21). Collectively, these studies support the notion that for interventions to be successful in the short and long term, they need to be relevant to the population being observed.

Strengths of this study include its being a randomized controlled trial with a pre, post, and one-year follow up design that targeted Hispanic adolescents, a group at increased risk for obesity. Limitations include the lack of a no-treatment control condition, though practical considerations and school requirements made this unfeasible. Although being able to randomize students at the individual level is a strength of the study, the randomization does not control for the possibility of contamination. Steps were taken to prevent contamination. All students assigned to a particular condition had identical class schedules so that they had class only with students also randomized to the same intervention condition. Although students ate lunch by grade level, students had assigned tables for lunch so that they ate lunch only with students randomized to the same intervention condition. It was not feasible to keep students separated according to condition during free times or extracurricular activities, and it is probable that those in the condition without *compañeros* knew that there was another condition and vice versa. Lastly, the health outcomes of *compañeros* were not assessed. Results from other studies that have measured the effects of peer health mentorship on the mentor suggest that health mentorship programs have health benefits for both parties involved (8).

More research is needed in the area of maintenance and translation of effective interventions for the school setting. School health initiatives are often deprioritized because of the pressures schools are under for students to perform well on standardized tests and because of resource constraints (22). Low-cost strategies that require little additional effort from the school’s staff are needed for school-based health programs to be sustainable. The findings of this study indicate that the addition of *compañeros* to an obesity program was an effective strategy among Hispanic adolescents to facilitate sustained reductions in zBMI for a year. Considering the effectiveness *compañeros* demonstrated in this study and the minimal extra resources needed to support them, the *compañeros* model warrants further investigation as a possible strategy for addressing practical concerns schools face when implementing health initiatives.
